# Supplementary material for: HNF1A recruits KDM6A to activate differentiated acinar cell programs that suppress pancreatic cancer
Source: EMBO J. 2020 Mar 10;39(9):e102808. doi: 10.15252/embj.2019102808 (PMC7196917; doi:10.15252/embj.2019102808)
Supplement: Supplementary file 1 — Appendix [file EMBJ-39-e102808-s001.pdf]

Appendix

HNF1A recruits KDM6A to activate a differentiated acinar cell program that suppresses pancreatic cancer

Table of contents

Appendix Figure S1 page 2

Appendix Figure S2 page 3

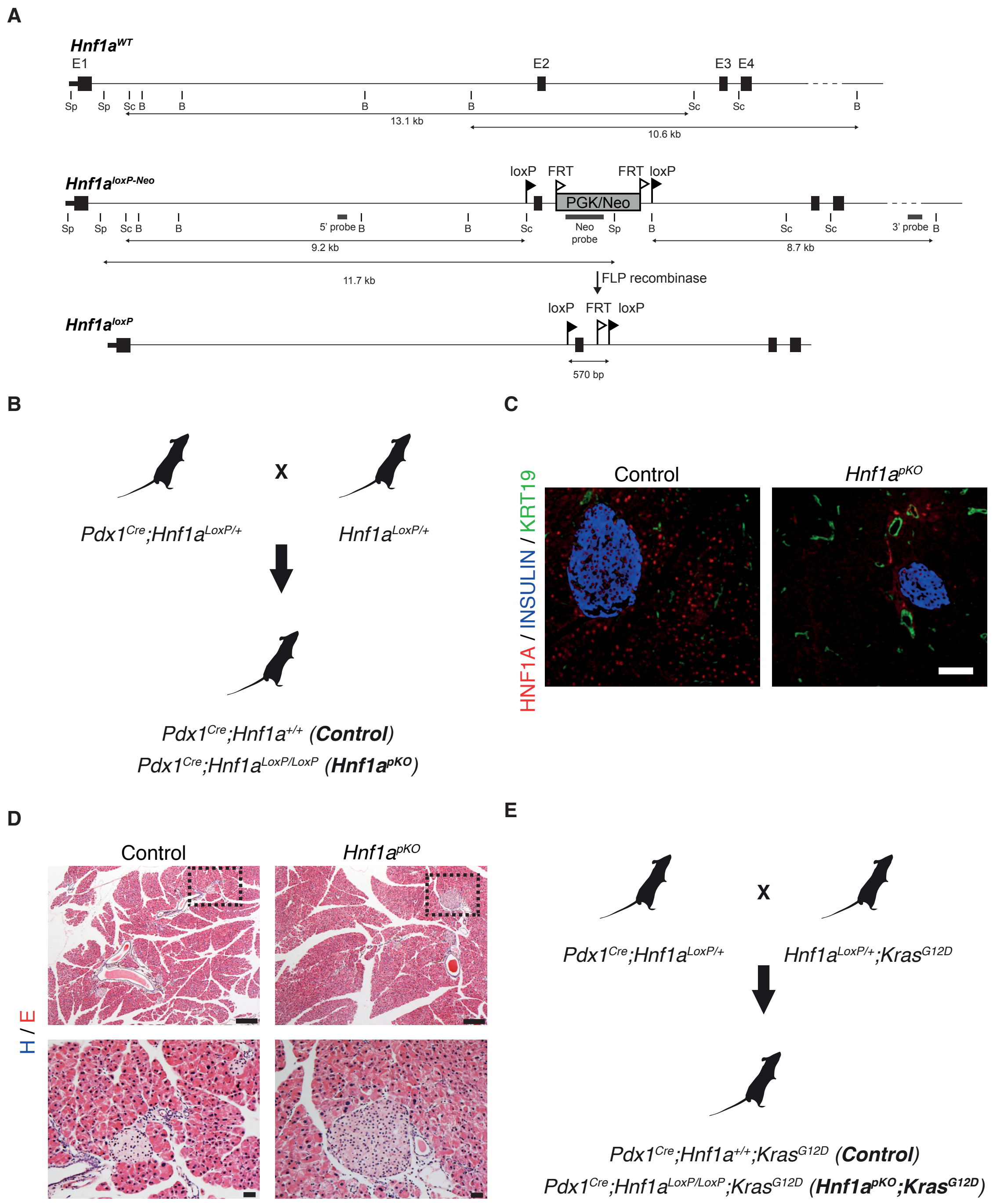

Appendix Figure S1. Strategy for creating a conditional *Hnf1a* mutant mouse line expressing *Kras*^{G12D}. A. Schematic of the targeted *Hnf1a* locus and the resulting *floxed exon 2* alleles, before and after excision of the PGK/Neomycin cassette by FLP-FRT recombination. B. Strategy for generating *Hnf1a*^{pKO} and control *Pdx1*^{Cre}*; Hnf1a*$^{+/+}$ mice. C. Immunofluorescence analysis shows that HNF1A (red) was efficiently excised in most acinar and endocrine cells from adult *Hnf1a*^{pKO} mice. Scale bar represents 50 µm. D. H&E staining showing normal pancreatic histology in *Hnf1a*^{pKO} mice, Scale bars represents 100 µm and 20 µm. e, Strategy for generating *Hnf1a*^{pKO}*;Kras*^{G12D} and control *Pdx1*^{Cre}*;Hnf1a*$^{+/+}$*; Kras*^{G12D} mice. B, BamHI; Sc, ScaI; Sp, SpeI.

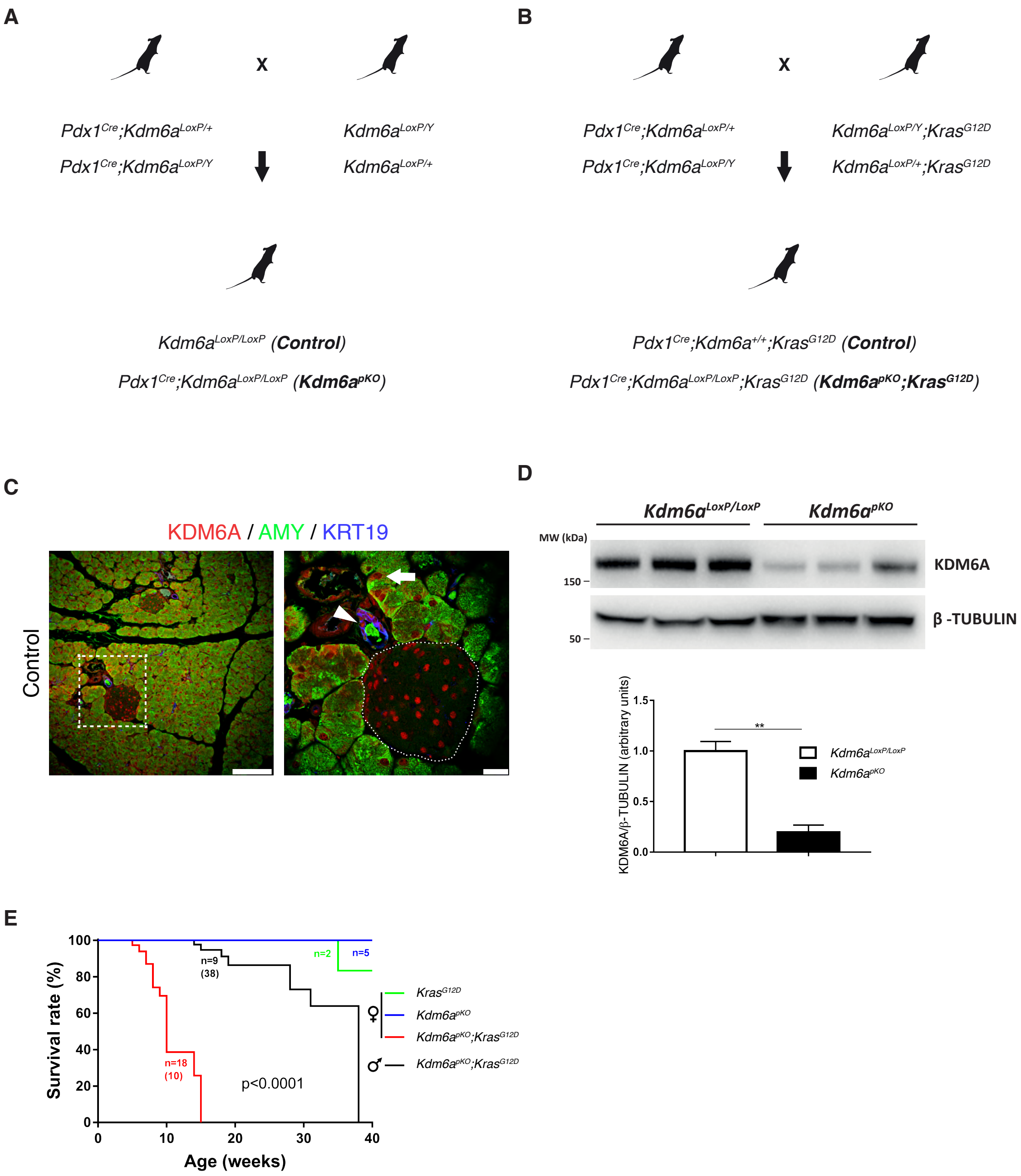

Appendix Figure S2. Pancreatic *Kdm6a* mutant mice expressing *Kras^{G12D}*. A-B. Schematic of strategy for generating *Kdm6a^{pKO}*, *Kdm6a^{pKO}*;*Kras^{G12D}* and control mice. C. Immunoblot showing reduced expression of KDM6A and average quantification of KDM6A relative to β-tubulin in pancreas from *Kdm6a$^{LoxP/LoxP}$* and *Kdm6a^{pKO}* mice, ** p<0.01, two tailed Student´s t test. D. Immunofluorescence analysis of KDM6A expression in wild type pancreas shows that KDM6A (red) is expressed in amylase positive (green) acinar cells, cytokeratin 19 positive (blue) ductal cells and in Islets of Langerhans (demarcated by dotted line). Scale bars represents (left) 100 µm and (right) 20 µm. E. Kaplan-Meier plot showing the survival of female *Kras^{G12D}*, *Kdm6a^{pKO}* and male and female *Kdm6a^{pKO}*;*Kras^{G12D}* mice, n= number of mice and numbers in brackets show median survival. Group median survival shown in brackets was compared using the Log-rank (Mantel-Cox) test.
