# Supplementary material for: HNF1A recruits KDM6A to activate differentiated acinar cell programs that suppress pancreatic cancer
Source: EMBO J. 2020 Mar 10;39(9):e102808. doi: 10.15252/embj.2019102808 (PMC7196917; doi:10.15252/embj.2019102808)
Supplement: Supplementary file 2 — Expanded View Figures PDF [file EMBJ-39-e102808-s002.pdf]

Expanded View Figures

Figure EV1. *Hnf1a* deletion leads to abnormal acinar cell identity and increased expression of genes related to oncogenic pathways.

A Breeding strategy to generate *Hnf1a*^{aKO} and *Ptf1a*^{Cre};*Hnf1a*$^{+/+}$ control mice using *Ptf1a*^{Cre} and *Hnf1a*^{LoxP} alleles.

B *Ptf1a*^{Cre} deletes HNF1A efficiently in acinar cells but to a lesser extent in islets of Langerhans. HNF1A IHC and hematoxylin staining in pancreas of control and *Hnf1a*^{aKO} mice. HNF1A is expressed in acinar and islet cells, but not in ductal cells in normal pancreas (left). HNF1A expression is depleted in acinar cells but largely not in islets in *Hnf1a*^{aKO} pancreas (right). The squared dotted boxes (top) indicate magnified areas (bottom). Arrows point at ducts, arrow head at HNF1A-positive acinar cell, and open arrow head at HNF1A positive islet cell. The dotted encircled areas indicate islets of Langerhans. Scale bar (top) 300 μm (bottom) 50 μm.

C H&E stainings in pancreas of control (left) and *Hnf1*^{aKO} mice (right) showing unaltered pancreatic morphology. Scale bar 300 μm.

D Expression of acinar differentiation genes in pancreas from *Hnf1a*^{aKO} and controls, depicted as box plots with median values and IQR of TPM values. Whiskers extend to highest and lowest data points within 1.5× IQR outside box limits. *P*-values were determined by two-tailed Student's *t*-test and n = 3 replicates per condition.

E GSEA showing increased expression of oncogenic pathways in *Hnf1a*^{aKO} pancreas.

F Western blots (top) and quantifications (bottom) showing increased phospho-p42 levels in *Hnf1a*^{aKO} pancreas. Quantification of signal intensities of phospho-p44/p42 normalized to total-p44/p42 levels. Data are shown as dots with mean and error bars ± SD. *P*-values were determined by two-tailed Student's *t*-test.

G Distribution of pancreatic HNF1A binding sites in annotated genomic regions.

H Venn diagrams illustrating that HNF1A-bound regions are enriched in regions of active promoters and enhancers. *P*-values and odds ratios were calculated by Fisher's exact test.

I Enrichment of known HNF1 motifs in the top 500 most significant HNF1A-bound ChIP-seq regions and percentage of regions containing each motif. The "union" is the percentage of regions with at least one motif sequence occurrence. Enrichment *P*-values are calculated using the one-tailed binomial test.

J Genome browser track for *Fn1* and *Timp1* genes showing upregulated expression in *Hnf1a*^{aKO} and *Kdm6a*^{pKO} pancreas, and absence of HNF1A or KDM6A binding in adjacent regions. Plots show TPM values normalized to *Hprt* with mean and error bars ± SD. *P*-values were determined by two-tailed Student's *t*-test.

 © 2020 The Authors

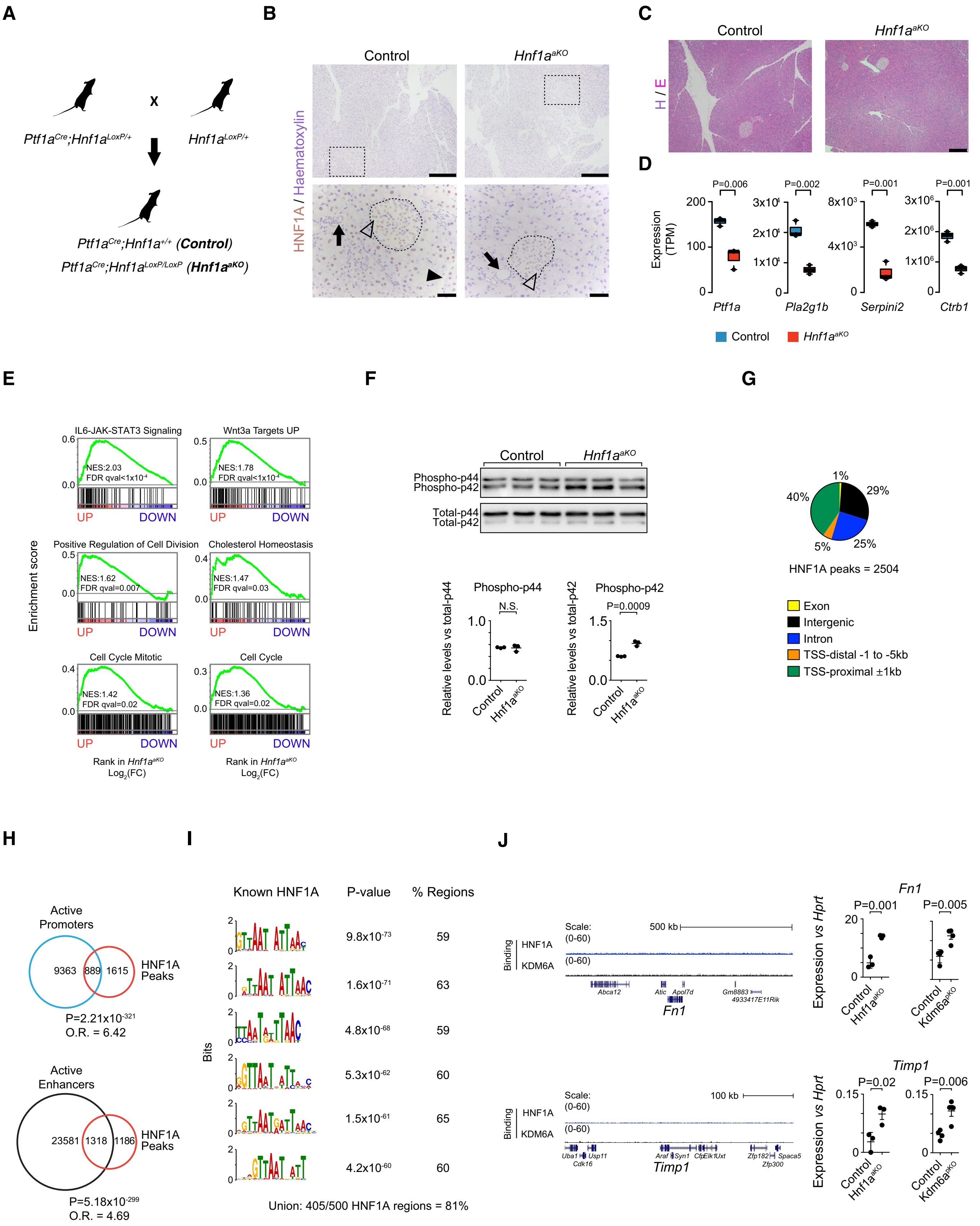
A
B
C
D
E
F
G
H
I
J
Ptf1aCre;Hnf1aLoxP/+
x
Hnf1aLoxP/+
Ptf1aCre;Hnf1a+/+ (Control)
Ptf1aCre;Hnf1aLoxP/LoxP (Hnf1aaKO)
Control
Hnf1aaKO
HNF1A / Haematoxylin
H / E
Expression (TPM)
P=0.006
P=0.002
P=0.001
P=0.001
Ptf1a
Pla2g1b
Serpini2
Ctrb1
Control
Hnf1aaKO
IL6-JAK-STAT3 Signaling
NES:2.03
FDR qval<1x10-4
Wnt3a Targets UP
NES:1.78
FDR qval<1x10-4
Positive Regulation of Cell Division
NES:1.62
FDR qval=0.007
Cholesterol Homeostasis
NES:1.47
FDR qval=0.03
Cell Cycle Mitotic
NES:1.42
FDR qval=0.02
Cell Cycle
NES:1.36
FDR qval=0.02
Enrichment score
UP
DOWN
Rank in Hnf1aaKO Log2(FC)
Phospho-p44
Phospho-p42
Total-p44
Total-p42
Relative levels vs total-p44
Relative levels vs total-p42
N.S.
P=0.0009
1%
29%
40%
5%
25%
HNF1A peaks = 2504
Exon
Intergenic
Intron
TSS-distal -1 to -5kb
TSS-proximal ±1kb
Active Promoters
9363
889
1615
HNF1A Peaks
P=2.21x10-321
O.R. = 6.42
Active Enhancers
23581
1318
1186
P=5.18x10-299
O.R. = 4.69
Known HNF1A
P-value
% Regions
Bits
9.8x10-73 59
1.6x10-71 63
4.8x10-68 59
5.3x10-62 60
1.5x10-61 65
4.2x10-60 60
Union: 405/500 HNF1A regions = 81%
Binding
HNF1A
KDM6A
Scale:
500 kb
100 kb
(0-60)
Fn1
Timp1
Expression vs Hprt
P=0.001
P=0.005
P=0.02
P=0.006
Control
Hnf1aaKO
Kdm6apKO

Figure EV1.

Figure EV2. *HNF1A* signature correlates with non-classical PDAC subtypes.

A Consensus clustered Z-score-normalized gene expression heatmaps of high-purity TCGA-PAAD and ICGC-PACA-AU human PDAC samples. Clustering was performed with non-negative matrix factorization based on expression of significantly down- and upregulated genes in *Hnf1a*^{aKO} pancreas. This revealed a cluster (HNF1A cluster 3) with concordant up- and downregulation of genes in *Hnf1a*^{aKO} pancreas, which predominantly matched non-classical PDAC molecular subtypes (quasimesenchymal, basal, squamous-like, pink in top tracks), as opposed to classical PDAC subtypes (green in top tracks). Multiclass SAM differentially expressed genes ($q < 0.05$) between HNF1A clusters are shown. Genes were hierarchically clustered using complete linkage with one minus Pearson correlation metrics. Along the right side of the heatmaps are green and red indicators of down- and upregulated genes in *Hnf1a*^{aKO} pancreas, respectively.

B *TP63* expression was increased in HNF1A LoF tumors compared to control tumors. RSEM normalized count data are shown as box plots with interquartile range, median, and whiskers. Box limits indicate the first and third quartiles and whiskers extend to highest and lowest data points within 1.5× IQR outside box limits. *HNF1A* LoF (n = 26), *Control 1* (n = 39), and *Control 2* (n = 57) tumors (P, Kruskal–Wallis).

C, D Expression of *HNF1A* and *KDM6A*, showing downregulation in non-classical PDAC subtypes (P, Kruskal–Wallis). Dots are RSEM normalized values presented with mean ± SD. Collisson subtypes: Quasimesenchymal (QM, n = 34) and Classical (CL, n = 54). Moffitt subtypes: Basal (BA, n = 65) and Classical (CL, n = 85). Bailey subtypes: Squamous-like (SQ-like, n = 31) and Pancreatic Progenitor (PP, n = 53).

E, F HNF1A levels are not lower in high histological grade PDAC (E), while KDM6A levels are (F). To determine whether histological grade of human PDAC was associated with expression levels of HNF1A (E) or KDM6A (F) proteins, we evaluated contingency tables of tumor grades versus staining intensities of each case in tissue microarray (TMA) IHC. Tumor grades were scored as either moderately differentiated (G2) or poorly differentiated/high grade (G3), and staining intensities were expressed as an Immuno Reactivity Score (IRS) reflecting either No, Weak, Moderate, or Strong staining intensities (see material and methods for details). Numbers of cases and percentages (in brackets) out of total cases are indicated for each tumor grade and staining intensity. The Chi-squared test was used to determine the probability of a significant relationship. Chi-square and P-values are shown. N = 217 patients for HNF1A and N = 208 patients for KDM6A.

 © 2020 The Authors

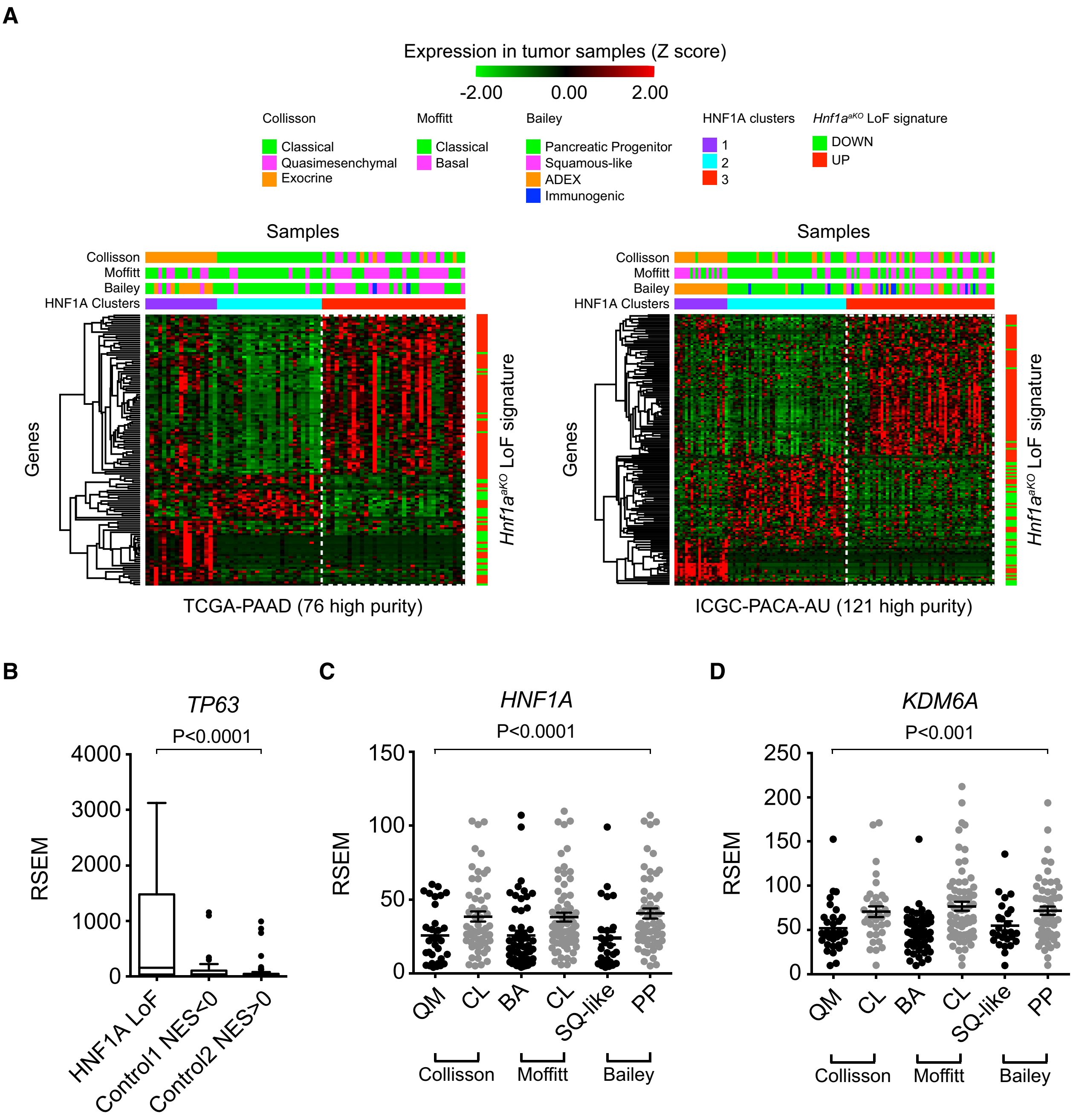

E

Tumor grade vs % HNF1A IRS

Grade	No	Weak	Moderate	Strong
G2	10 (4.6%)	31 (14.3%)	65 (30%)	9 (4.1%)
G3	4 (1.8%)	37 (17.1%)	53 (24.4%)	8 (3.7%)

Chi-Square total = 3.6142
P-Value = 0.306253

F

Tumor grade vs % KDMA6A IRS

Grade	No	Weak	Moderate	Strong
G2	45 (21.6%)	35 (16.8%)	32 (15.4%)	2 (0.9%)
G3	50 (24.1%)	32 (15.4%)	11 (5.3%)	1 (0.5%)

Chi-Square total = 9.1481
P-Value = 0.027385

Figure EV2.

© 2020 The Authors

Figure EV3. KDM6A suppresses growth and oncogenic pathways and maintains acinar cell integrity.

A Efficient deletion of *Kdm6a* in the pancreatic epithelium at E15.5. KDM6A (red) is ubiquitously expressed in all pancreatic cells. CDH1 (green) marks epithelial cells. Upon deletion, KDM6A staining is lost specifically in CDH1-expressing epithelial cells but not in mesenchymal cells or in the stomach epithelium (white arrow heads). Scale bar indicates 100 μm.

B *Kdm6a* mutant mice show normal fasting and fed glycemia. The horizontal stroked line indicates blood glucose levels at 250 mg/dl as a reference.

C–H The pancreas of *Kdm6a*^{pKO} mice were histologically normal until 8 weeks of age. At 8 weeks of age, some signs of acinar cell attrition and fat replacement could be observed, as shown in this representative image. Scale bars: 250 μm (10× magnification), 50 μm (40× magnification).

I Representative picture (left) showing increased number of KI67 (red) amylase-expressing acinar cells (green) in *Kdm6a*^{pKO} pancreas. Scale bar, 250 μm. Quantifications (right) were performed on three pancreatic sections separated by at least 100 μm from 4 control and 4 *Kdm6a*^{pKO} mice. Acinar cell proliferation was represented as the average of the KI67^{+}/Amylase^{+} cell ratio ± SD. *P*-values were determined by two-tailed Student's *t*-test.

J GSEA plots showing enrichment of Oncostatin M and "TNFA signaling via NFKB" gene sets among genes upregulated in *Kdm6a*^{pKO} pancreas.

K Western blots (top) and quantifications (bottom) showing increased phospho-p44/p42 levels in *Kdm6a*^{pKO} pancreas. Quantification of signal intensities of phospho-p44/p42 normalized to total-p44/p42 levels. Data are shown as dots with mean and error bars ± SD. *P*-values were determined by two-tailed Student's *t*-test.

L Most significantly deranged REACTOME pathways in both KDM6A- and HNF1A-deficient pancreas (see also Dataset EV5).

M *Kdm6a*^{pKO} down- and upregulated gene sets showed concordant deregulation in *KDM6A* LoF mutant tumors versus classical PDAC (based on Bailey *et al*'s signature) (Bailey *et al*, 2016).

N Tumors with KDM6A-deficient phenotypes showed decreased *KDM6A* mRNA. We created a gene set of human orthologs of *Kdm6a*^{pKO} downregulated genes, and for each high-purity tumor sample in the ICGC-PACA-AU, we used GSEA to test for enrichment of this gene set in gene lists that were rank-ordered by differential expression in the individual sample versus all other samples. Samples with NES < 0 and *P*-value < 0.05 were considered as having KDM6A LoF phenotypes and were compared against all other samples. *Z*-score-normalized count data are shown as box plots with IQR, median, and whiskers. Whiskers extend to highest and lowest data points within 1.5× IQR outside box limits. *P*-values were determined by two-tailed Student's *t*-test.

O Gene sets that showed up- or downregulation in non-classical human PDAC showed concordant enrichment in up- or downregulated genes in *Kdm6a*^{pKO} versus control pancreas. GSEA NES and FDR q-values are shown.

P Genomic distribution of KDM6A binding sites in mouse pancreas.

Q, R Top: ChIP-seq and RNA-seq tracks in control and *Kdm6a*^{pKO} pancreas, in two loci harboring downregulated genes (*Kif12*, *Gprc5c*) in *Kdm6a*^{pKO} pancreas. Bottom: ChIP-qPCR validations for regions highlighted in green (R1, R2, R3), showing that *Kdm6a* mutants have increased H3K27me3 and decreased H3K27ac in most regions. H3K4me1 was also decreased in mutants in distal sites. Error bars show SD, and *P*-values were determined by two-tailed Student's *t*-test, $n = 3$.

S KDM6A-bound regions are enriched in active pancreas promoters and enhancers. *P*-values are calculated by Fisher's exact test.

T, U Genome Browser examples (top) of HNF1A and KDM6A binding to genes known as negative regulators of EMT: *Gstp1* (T) and *Deptor* in (U) that are downregulated in *Hnf1a*^{aKO} and *Kdm6a*^{pKO} pancreas (bottom). Plots show TPM values normalized to *Hprt* with mean and error bars ± SD. $N = 4$ per condition and *P*-values were determined by two-tailed Student's *t*-test.

 © 2020 The Authors

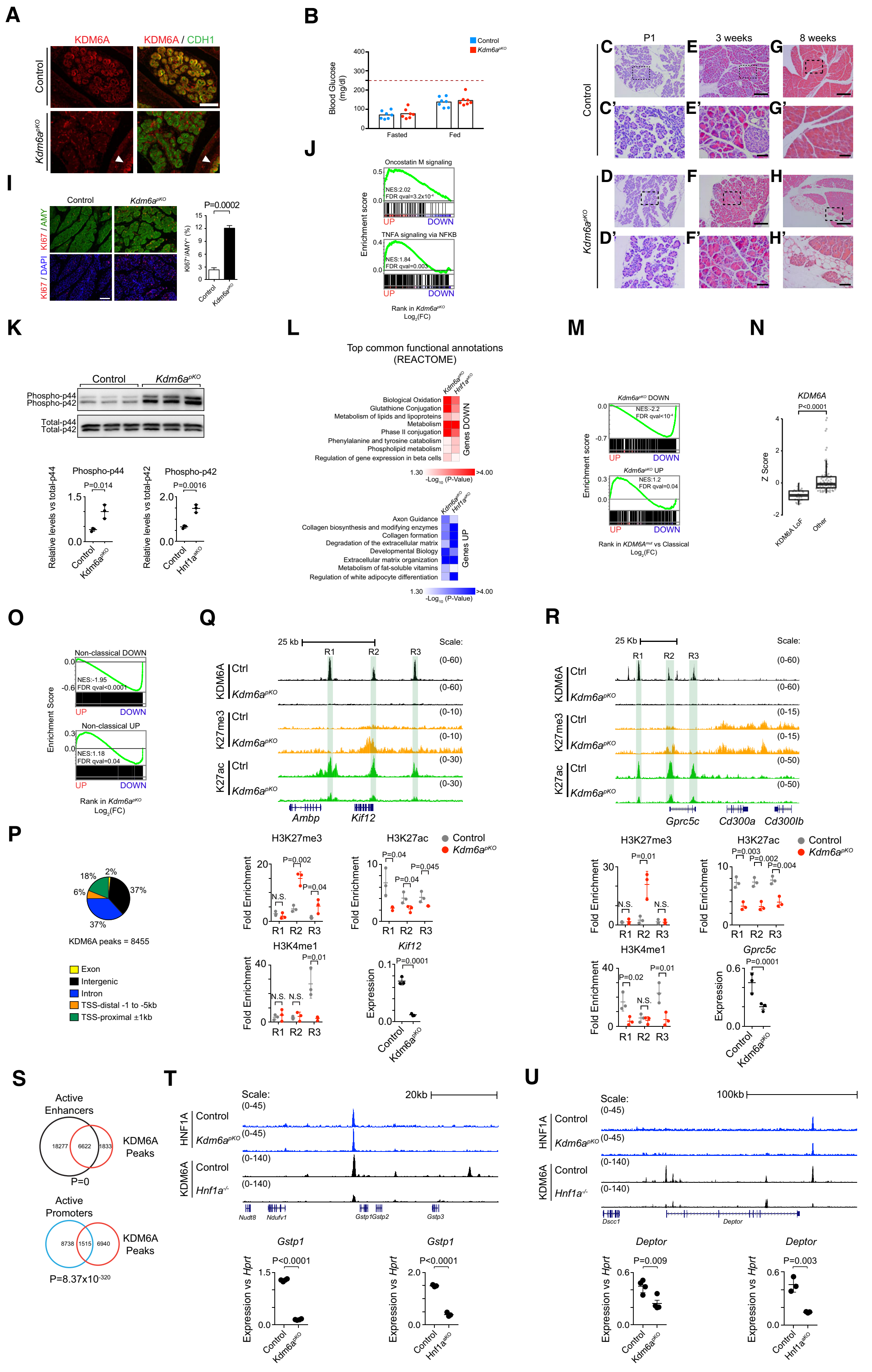

Figure EV3.

Figure EV4. KDM6A binding to target genes is dependent on HNF1A, while HNF1A can bind to chromatin in the absence of KDM6A.

A–D Left: Genome Browser examples of loci co-bound by HNF1A and KDM6A, showing loss of KDM6A binding in HNF1A-deficient pancreas (region highlighted in green) and decreased RNA levels in HNF1A-deficient pancreas. Right: ChIP-qPCRs showing loss of KDM6A and HNF1A binding in highlighted regions in left and qPCRs show downregulation of target genes in *Hnf1a*-KO pancreas. Error bars show SD, and *P*-values were determined by two-tailed Student's *t*-test, $n = 4$ for ChIP-qPCRs and $n = 3$ for qPCRs.

© 2020 The Authors

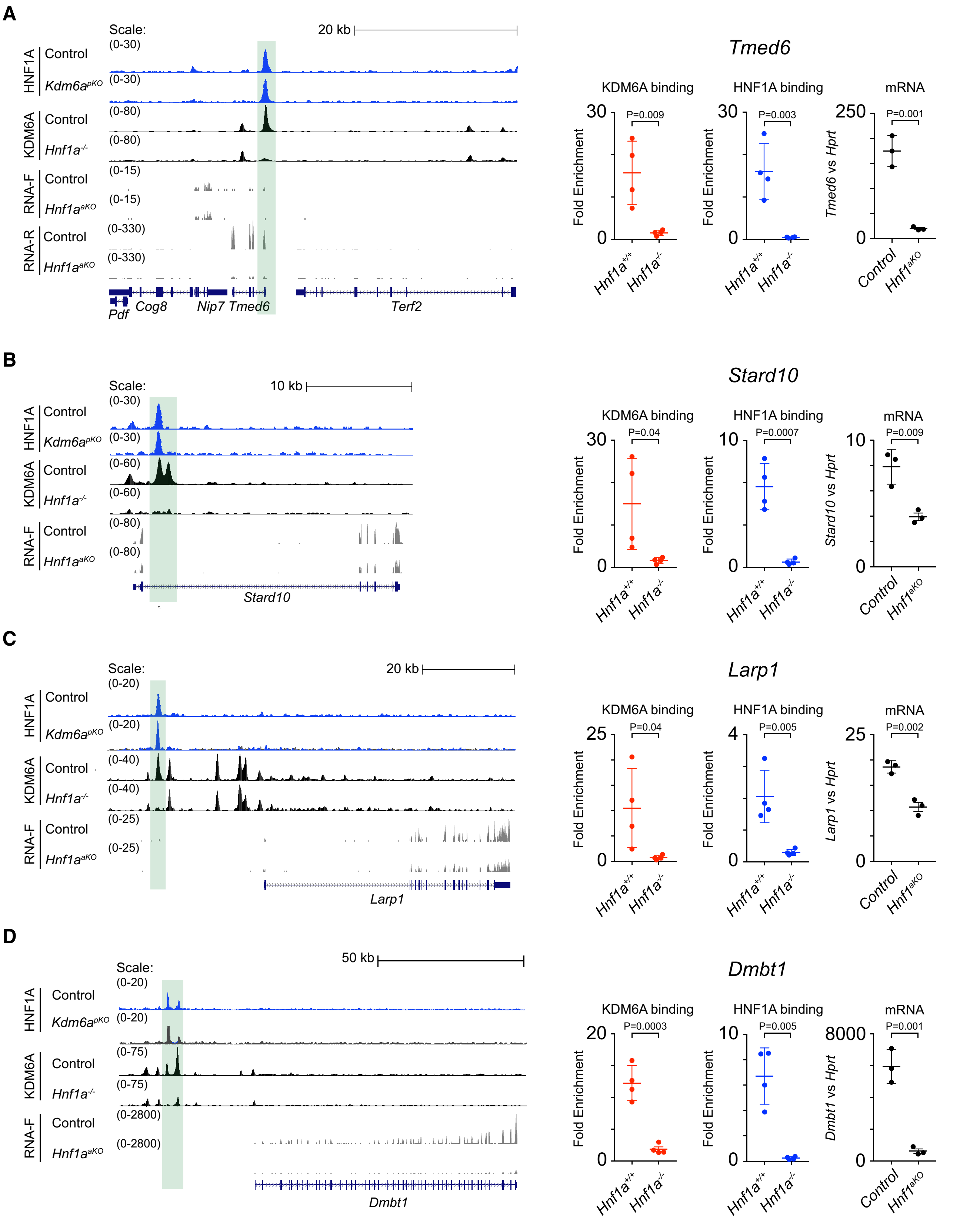
A
Scale:
20 kb
HNF1A
Control
Kdm6apKO
KDM6A
Control
Hnf1a-/-
RNA-F
Control
Hnf1aaKO
RNA-R
Control
Hnf1aaKO
(0-30)
(0-80)
(0-15)
(0-330)
Pdf
Cog8
Nip7 Tmed6
Terf2
Tmed6
KDM6A binding
HNF1A binding
mRNA
Fold Enrichment
Tmed6 vs Hprt
P=0.009
P=0.003
P=0.001
Hnf1a+/+
Hnf1a-/-
Control
Hnf1aKO
B
Scale:
10 kb
(0-30)
(0-60)
(0-80)
Stard10
KDM6A binding
HNF1A binding
mRNA
P=0.04
P=0.0007
P=0.009
Stard10 vs Hprt
C
Scale:
20 kb
(0-20)
(0-40)
(0-25)
Larp1
KDM6A binding
HNF1A binding
mRNA
P=0.04
P=0.005
P=0.002
Larp1 vs Hprt
D
Scale:
50 kb
(0-20)
(0-75)
(0-2800)
Dmbt1
KDM6A binding
HNF1A binding
mRNA
P=0.0003
P=0.005
P=0.001
Dmbt1 vs Hprt

Figure EV4.

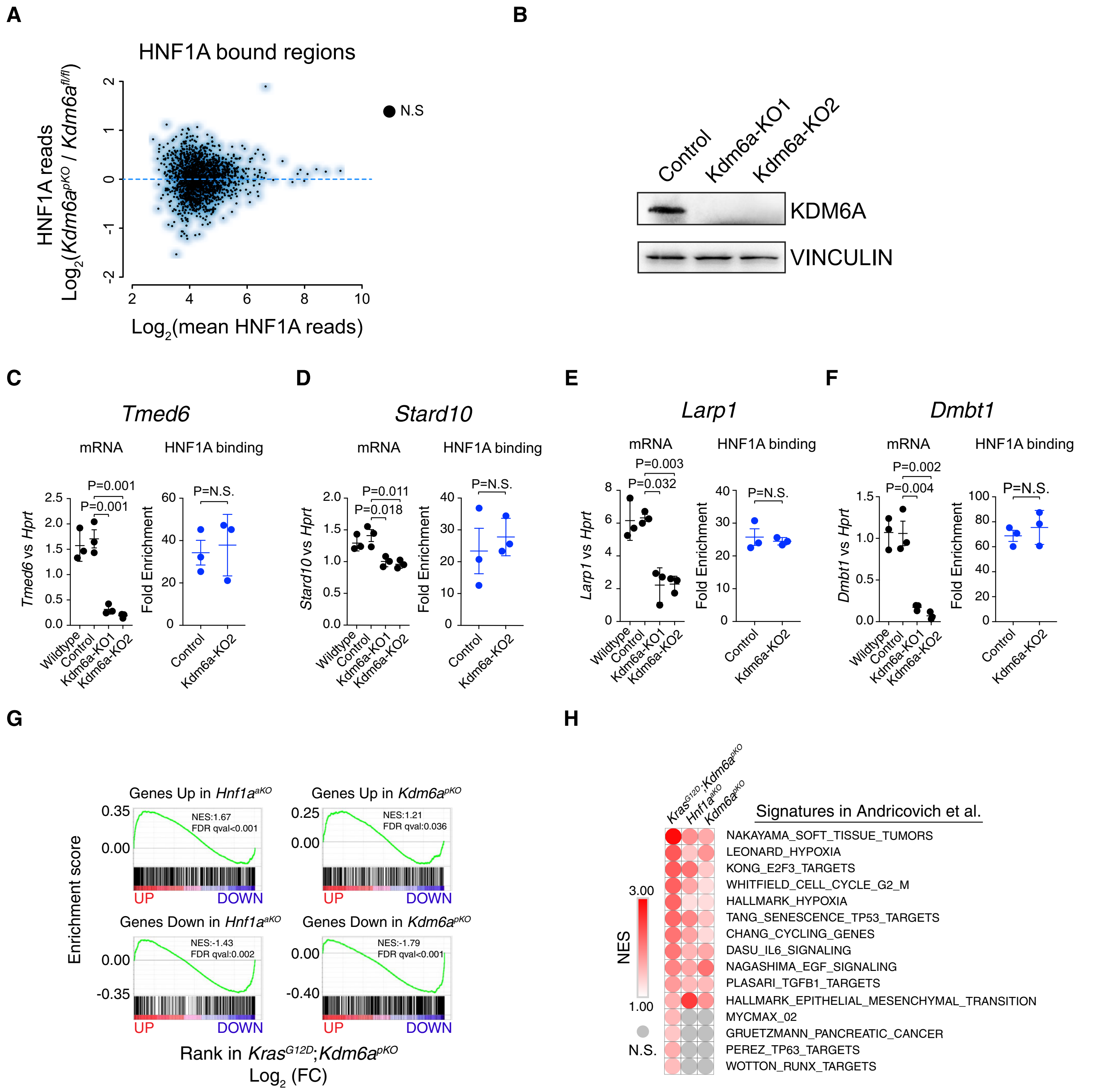

Figure EV5. KDM6A dependency of HNF1A-mediated transcription.

A HNF1A binding to chromatin is unaffected in *Kdm6a*^{pKO} pancreas. Scatterplot showing unchanged HNF1A binding in *Kdm6a*^{pKO} versus control pancreas (e.g., see also Appendix Fig S6A–D).

B Western blot showing KDM6A depletion in two clones from *Kdm6a*-KO acinar cell lines.

C–F qPCR in *Kdm6a*-KO acinar cell lines (KO1 and KO2) shows reduced expression of HNF1A bound genes, while ChIP-qPCRs for HNF1A show that its binding to those genes is unchanged when KDM6A is depleted. Selected genes and HNF1A binding regions were from Appendix Fig S6A–D. qPCR data are relative mRNA expression of indicated genes versus *Hprt*. ChIP-qPCR values indicate fold enrichment relative to control region. Error bars show ± SD, and *P*-values were determined by two-tailed Student's *t*-test.

G GSEA analysis on ranked-ordered gene list from *Kras*^{G12D};*Kdm6a*^{pKO} data from Andricovich *et al* (2018) versus gene sets from up- or downregulated genes in *Hnf1a*^{aKO} and *Kdm6a*^{pKO} pancreas demonstrates that KDM6A and HNF1A regulate similar genes in normal and *Kras*^{G12D}-transformed pancreas.

H GSEA, comparing rank-ordered expression data from *Kras*^{G12D};*Kdm6a*^{pKO}, *Hnf1a*^{aKO}, and *Kdm6a*^{pKO} mice with gene sets from Andricovich *et al* (2018), shows that most of the pathways that are enriched in *Kdm6a*-deficient pancreatic cancer are dependent on HNF1A and KDM6A function in the non-tumoral pancreas.

 © 2020 The Authors
